# Acute heart failure associated with toxic shock syndrome due to methicillin-susceptible *Staphylococcus aureus* during the postpartum period: case report and systematic literature review

**DOI:** 10.1186/s12872-022-02903-3

**Published:** 2022-10-29

**Authors:** Takahiro Suzuki, Takahiro Matsuo, Yasufumi Kijima, Ryo Hasegawa, Kazuhiro Ishikawa, Michiko Yamanaka, Fujimi Kawai, Nobuyuki Komiyama, Nobuyoshi Mori

**Affiliations:** 1 Department of Cardiovascular Medicine, St. Luke’s International Hospital, Tokyo, Japan; 2 Department of Infectious Diseases, St. Luke’s International Hospital, Tokyo, Japan; 3 Department of Integrated Women’s Health, St. Luke’s International Hospital, 9-1 Akashicho, Chuo-ku, Tokyo, 104-8560 Japan; 4 St. Luke’s International University Library, Tokyo, Japan

**Keywords:** Acute heart failure, Toxic shock syndrome, Postpartum, *Staphylococcus aureus*

## Abstract

**Background:**

Toxic shock syndrome (TSS) caused by *Staphylococcus aureus* in the postpartum period is a rare but life-threatening disease. We present a case of acute heart failure as the initial presentation of TSS due to methicillin-susceptible *Staphylococcus aureus* (MSSA) and describe its clinical characteristics with a systematic literature review.

**Case presentation:**

A 34-year-old woman, 8 days after a normal vaginal delivery presented to our hospital with dyspnea and fever. She had jugular venous distension, bilateral leg edema, and erythema. Laboratory examinations revealed elevated NT-pro-BNP level of 3,233 pg/mL. Transthoracic echocardiography showed elevated tricuspid regurgitation peak gradient, with decreased respiratory variability of the inferior vena cava diameter and bilateral pleural effusions. The patient was hospitalized with suspicion of congestive heart failure. MSSA positive for toxic shock syndrome exotoxin-1 was detected in the culture of the perineal incision wound, and we diagnosed TSS caused by MSSA. Intravenous diuretics were administered, along with eventual cefazolin plus clindamycin. After 2 weeks of antimicrobial therapy, the patient showed improvement and was discharged. No recurrence was observed at the 24-month follow-up.

**Conclusion:**

This is a rare case report of acute heart failure being the initial manifestation of TSS due to MSSA in the postpartum period. Clinicians should consider TSS as a possibility in postpartum patients with acute heart failure. This systematic review provides insights into its clinical features, treatment regimens, and prognosis of TSS by *S. aureus* in the postpartum period. TSS requires an appropriate, prompt diagnosis, because delayed treatment can be fatal.

**Supplementary Information:**

The online version contains supplementary material available at 10.1186/s12872-022-02903-3.

## Background

Toxic shock syndrome (TSS) is a syndrome caused by toxic shock syndrome toxin-1 (TSST-1), an exotoxin produced by *Staphylococcus aureus* (*S. aureus*), which leads to various severe clinical symptoms, such as generalized erythema, hypotension, septic shock, and multiple organ failure [1]. As Todd et al. first reported TSS in 1978, TSS is a rare but life-threatening disease, and it has later been classified into menstruation-related and non-menstruation-related types [2]. Ever since Whitefield et al. reported TSS in the postpartum period in 1981, non-menstrual TSS has been increasingly reported [3]. Although there are diagnostic guidelines published by the Centers for Disease Control and Prevention (CDC) for TSS, early diagnosis is difficult because of the diversity of its symptoms [4]. Therapeutic intervention is often delayed by misdiagnosis, emphasizing the importance of awareness of this disease among healthcare professionals [5].

Heart failure during the postpartum period is an extremely uncommon manifestation of TSS, as noted in the present case. To clarify the clinical characteristics of TSS caused by *S. aureus* during the postpartum period, we conducted a systematic literature review to determine the prognosis, complication rate of heart failure, and treatment strategy. Herein, we present the case report of acute heart failure as the initial presentation of TSS due to methicillin-susceptible *Staphylococcus aureus* (MSSA) and describe its clinical characteristics in a systematic review of TSS due to *S. aureus* in the postpartum period.

## Case presentation

**Fig. 1:**
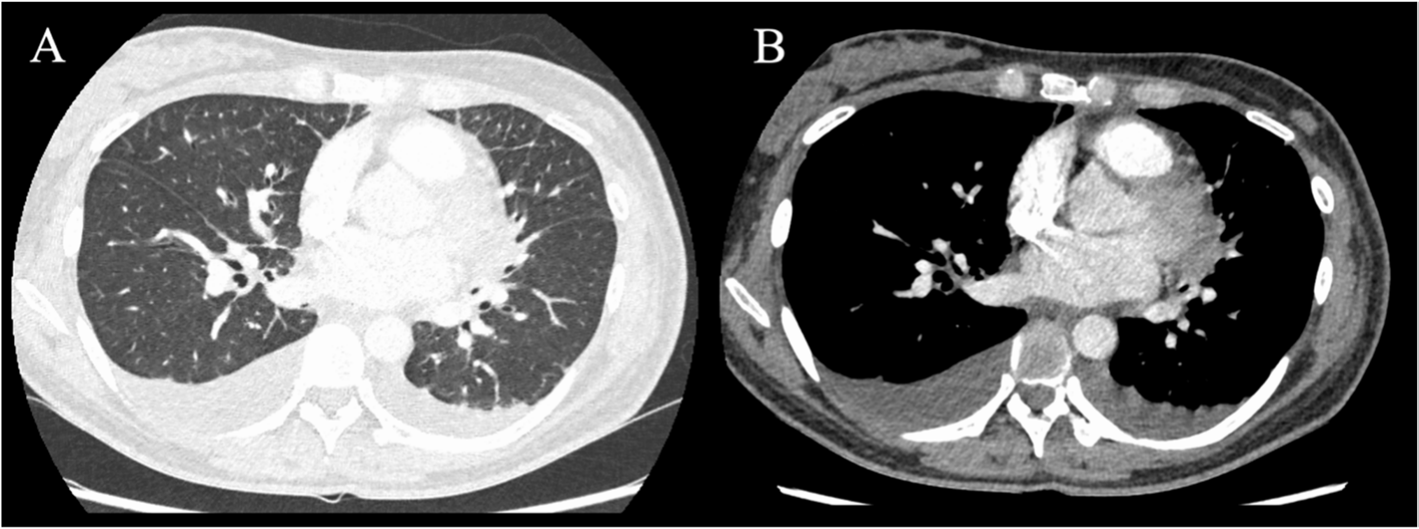
Computed Tomography on arrival at the hospital. **A** The chest CT (non-contrast) revealed bilateral pleural effusions, interlobular septal thickening in both lungs, and ground glass opacification in both lungs. The findings are consistent with pulmonary edema. **B** Contrast-enhanced CT showed no contrast defect in the pulmonary artery and only an enlarged uterus due to postpartum. There were no abnormal findings in other organs. CT, Computed Tomography

A 34-year-old woman without significant past medical history presented to our emergency department with acute onset of dyspnea three days prior. Eight days prior to admission, she delivered vaginally at a gestational age of 39 weeks. Her pregnancy progress was fairly good, except she had a huge (goose-egg size) perineal varicose veins on her right side, therefore episiotomy was performed on her left side considering the risk of rupture of varicose. She had no neither present nor past alcohol intake, smoking history, nor was she taking any regular medications. A 12-lead ECG 3 months prior to the delivery showed no abnormal findings. On admission, the patient was in mild distress and had a temperature of 37.1 °C, blood pressure of 123/74 mmHg, heart rate of 121/min, respiratory rate of 30/min, and oxygen saturation of 86% on room air. Physical examination revealed erythema on the face and trunk with bilateral lower leg pitting edema, jugular vein distension, perineal varicose veins, and redness with purulent discharge at the episiotomy site. Laboratory data showed hemoglobin of 9.1 g/dL with the mean corpuscular volume of 82.5 fL, white blood cell count of 16,200/μL (87% neutrophils, 4% lymphocytes, 6% monocytes, and 3% eosinophils), aspartate aminotransferase of 69 U/L, alanine aminotransferase of 64 U/L, lactate dehydrogenase of 304 U/L, C-reactive protein (CRP) of 26.9 mg/dL, creatine kinase-muscle-brain of 14 U/L, cardiac troponin T 0.037 ng/mL, and the N-terminal prohormone of brain natriuretic peptide of 3,233 pg/mL. Electrolytes and renal function were normal. Other diagnostic workups, including IgM antibodies for Coxsackie virus, echovirus, cytomegalovirus, adenovirus, Epstein-Barr virus, herpes simplex, and varicella-zoster virus, were all negative. A 12 lead ECG clearly demonstrated a normal sinus rhythm. Chest radiography revealed pulmonary venous congestion and Kerley's B-line in both the lungs. Contrast-enhanced computed tomography (CT) of the chest-abdomen-pelvis demonstrated bilateral pleural effusion and conspicuous interstitial thickening, a finding of pulmonary edema, and no signs of intrauterine or perineal abscess (Fig. 1). Transthoracic echocardiography (TTE) revealed unremarkable left ventricular size and wall thickness with a normal ejection fraction (62.4%; disk method). The echocardiographic four-chamber image is shown in Video 1, and a dilated inferior vena cava (IVC) of 21.7/18.4 mm (inspiration and expansion during expiration) is shown in Video 2. The findings of each measurement are summarized in Tables 1 and 2. Regarding parameters associated with left ventricular diastolic dysfunction, this patient had a septal eʼ < 7 cm/s, a peak tricuspid regurgitation velocity > 2.8 m/s, and a resting tricuspid regurgitation peak gradient (TRPG) of 41 mmHg. The left atrial volume index (LAVI) was 27 ml/m^2^, and the E/e' (average) was 12.6. These values corresponded to having an intermediate left ventricular diastolic dysfunction [6]. Furthermore, TTE showed no evidence of cardiac vegetations. The patient was admitted to the intensive coronary care unit on suspicion of infection-induced acute heart failure, postpartum cardiomyopathy, or viral myocarditis.

**Table 1:** Echocardiographic findings at initial admission and after clinical improvement

Echocardiographic Data	Day 1	Day 10
LVEF (%)	62.4	63.1
LVDd (mm)	51.1	52.3
LVDs (mm)	32.2	32.6
LAD (mm)	37.4	32.5
LAVI (mL/m^2^)	27	25
E (cm/sec)	105.3	84.4
A (cm/sec)	47.3	50
E/A	2.2	1.7
e’ (septal) (cm/s)	6.7	8
e’ (lateral) (cm/s)	11.2	16.9
E/e’	12.6	7.7
DcT (ms)	137	187
TRV (m/s)	3.2	2.6
RV basal (short axis) (mm)	39.7	38.4
RV mid (short axis) (mm)	34.4	33.6
RV (long axis) (mm)	69.4	68.7
RA (short axis) (mm)	32.1	30.1
RA (long axis) (mm)	42.5	40.1
IVC maximal diameter (mm)	21.7	19.3
IVC minimal diameter (mm)	18.4	7.2

*LVEF* Left ventricular ejection fraction, *LVDd* Left ventricular dimension end diastole, *LVDs* Left ventricular end-systolic diameter, *LAD* Left atrial dimension, *LAVI* Left atrial volume index, *DcT* Deceleration time, *TRV* Tricuspid regurgitation velocity, *RV* Right ventricular, *RA* Right atrium, *IVC* Inferior vena cava

**Table 2:** Characteristics of patients with toxic shock syndrome due to *Staphylococcus aureus* during the postpartum period

Case	Case Reference	Year of Report	Age	Gravidity / Parity	Underlying Diseases	Chief complaint	Delivery	Days after delivery	Entry of the Infection	Pathogen	Toxin	Complication of heart failure	Antimicrobial regimen	IVIg	Outcome
1	Sorrell	1981	28	G0P0	Vaginal moniliasis	Fever, chills	CS	2	SSI	MSSA	N/A	Yes	Oral amoxycillin 500 mg thrice, and metronidazole 200 mg thrice daily, followed by flucloxacillin IV 4 g for 14 days, metronidazole 500 mg thrice for 10 days and gentamicin for 14 days	None	N/A
2	Whitefield	1981	24	G1P0	None	Fever, nausea, vomiting, diarrhea, sore throat, headache, myalgia	VD	1	Endometritis	MSSA	TSST-1	No	Ampicillin 1 g IV every four hours for 2 days, followed by nafcillin 2 g IV every four hours	None	Cure
3	Green	1982	28	G2P2	None	Fever, nausea, vomiting, diarrhea, abdominal pain, sore throat, myalgia arthralgias	VD	1	Endometritis/episiotomy	MSSA	N/A	No	Cefoxitin and tobramycin, followed by dicloxacillin	None	Cure
4	Bracero	1982	25	G2P1	N/A	Fever, chills, diarrhea	VD	1	Perineum	MSSA	N/A	No	Ampicillin, followed by Cefazolin for 5 days	None	Cure
5	Cheek	1982	25	G2P1	None	Fever, lower abdominal pain	VD	2	N/A	MSSA	N/A	No	Methicillin	None	Cure
6	Guerinot	1982	24	G0P0	N/A	Fever, myalgia, nausea, vomiting, diarrhea	VD	1	Perineum	MSSA, β hemolytic Streptococcus	N/A	No	Ampicillin, kanamycin, followed by penicillin, clindamycin, kanamycin, followed by tetracycline, nafcillin, clindamycin	None	Cure
7	Chow	1984	28	G2P1	None	Fever, vomiting, generalized rash	VD	3	Endometritis/episiotomy	MSSA	Enterotoxin C	No	Cloxacillin 500 mg every 6 h orally for 5 days	None	Cure
8	Katoh	1988	29	G4P2	N/A	Fever, diarrhea, general myalgia, general erythema	VD	6	Endometritis	*S. aureus*	Enterotoxin C, TSST-1	No	Fosfomycin 2 g thrice, tobramycin 60 mg thrice, followed by minocycline	None	Cure
9	Derney	1989	23	G0P0	N/A	Fever, chills, myalgia, throat pain, nausea, vomiting, diarrhea, nausea, generalized itching, diffuse myalgia, pain in the right breast	N/A	8	Mastitis	MSSA	N/A	No	Erythromycin, followed by cloxacillin and amikacin	None	Cure
10	Kadoya	1996	32	N/A	SLE	Fever, diarrhea, erosive erosions of the vulva, sore throat, erythema of limbs	VD	2	Perineum	MRSA	TSST-1	No	Vancomycin	None	Cure
11	Gregg	1997	26	N/A	None	Fever, nausea, vomiting, diarrhea, mild soreness in the left breast	VD	8	Mastitis	*S. aureus*	N/A	No	Nafcillin, followed by vancomycin	None	Cure
12	Kisaka	1997	28	G0P0	N/A	Fever, diarrhea, erythema, myalgia	VD	2	Perineum	MRSA	N/A	No	Fosfomycin, levofloxacin, followed by minocycline, vancomycin, followed by amikacin, vancomycin, followed by sulfamethoxazole—trimethoprim, vancomycin for total 14 days	None	Cure
13	Ohashi	2000	37	G1P1	Diabetes mellitus, acromegaly, hypopituitarism	Fever, generalized erythema, dizziness	VD	1	N/A	MRSA	N/A	No	Flomoxef, followed by imipenem / cilastatin, followed by vancomycin for total 13 days	5000 mg/day for 3 days	Cure
14	Ohashi	2000	20	G0P0	None	Fever, sore throat, skin rash on lower extremities and abdomen, myalgia	VD	27	N/A	MRSA	N/A	No	Cefotiam, followed by amikacin, followed by vancomycin for total 7 days	None	Cure
15	Fujiwara	2001	23	G1P1	Febrile convulsion	Fever, nausea, vomiting, diarrhea, general malaise, generalized erythema, pruritus, myalgia in the abdomen and back, photophobia, eyelid conjunctiva hyperemia, fainting, oliguria	VD	21	Mastitis	MRSA	TSST-1, enterotoxin C	No	Cefteram pivoxil 300 mg/day, followed by vancomycin, imipenem / cilastatin, followed by arbekacin for total 21 days	None	Cure
16	Tanaka	2002	25	N/A	None	Fever, generalized erythema	VD	5	Perineum	MRSA	TSST-1	No	Cefdinir for 5 days, followed by flomoxef for 7 days	None	Cure
17	Andrews	2008	20	N/A	None	Fever, left breast tenderness, rash, chills	CS	28	Mastitis	MRSA	Enterotoxin B	No	Clindamycin, tazobactam / piperacillin, followed by clindamycin, vancomycin for total 14 days	None	Cure
18	Collet	2009	38	N/A	N/A	Fever, vomiting, diarrhea, skin rash, lower back pain at the left side	VD	28	Endometritis	MRSA	TSST-1	No	Cefotaxime, vancomycin, metronidazole, followed by vancomycin for total 10 days	None	Cure
19	Saito	2010	28	N/A	None	Fever, generalized erythema	CS	9	Endometritis	MRSA	N/A	No	Teicoplanin, clindamycin	5000 mg/day for 3 days	Cure
20	Ito	2011	32	N/A	N/A	Fever, generalized erythema, diarrhea	VD	8	Endometritis	MRSA	TSST-1	No	Cefcapene pivoxil, followed by ciprofloxacin, followed by vancomycin	None	Cure
21	Manabe	2016	36	N/A	None	Fever, general fatigue, diarrhea, extensive macular erythematous	CS	3	SSI	MRSA	TSST-1, enterotoxin C	No	Sulbactam / Ampicillin, clindamycin, followed by sulbactam / ampicillin, clindamycin, linezolid	None	N/A
22	Deguchi	2017	32	G4P3	None	Fever, focal perineal pain	VD	12	Perineum	MRSA	TSST-1	No	Ceftriaxone, clindamycin for 11 days	None	Cure
23	Gokaraju	2017	28	N/A	None	Fever, nausea, vomiting, diarrhea	VD	12	Mastitis	MSSA	N/A	No	Meropenem, clindamycin, vancomycin, followed by nafcillin, clindamycin	None	Cure
24	Masuko	2018	34	G6P2	None	Fever, nausea, vomiting, diarrhea, headache, and blisters on both upper limbs	CS	4	SSI	MRSA	TSST-1	No	Flomoxef, followed by ampicillin, clindamycin, vancomycin, micafungin, followed by, ampicillin, clindamycin, ampicillin, clindamycin,	5000 mg/day for 3 days	N/A
25	Shibata	2021	33	G0P0	None	Fever, uterine tenderness, lip edema, systemic erythema, epidermal detachment, diarrhea	CS	1	N/A	MRSA	N/A	No	Clindamycin, followed by teicoplanin and meropenem for total 12 days	None	Cure
26	Shibata	2021	26	G0P0	None	Fever, strawberry tongue, angular cheilitis, diarrhea, skin symptoms (redness and papules)	CS	4	Perineum	MRSA	N/A	No	Cefazolin, followed by sulbactam/ampicillin, followed by vancomycin for total 12 days	None	Cure
PR	Suzuki	2020	34	G0P0	None	Dyspnea, pitting edema, erythema, fever, diarrhea	VD	8	Perineum	MSSA	TSST-1	Yes	Ceftriaxone, metronidazole, ampicillin, clindamycin, followed by cefepime, metronidazole, clindamycin, followed by cefazolin for total 14 days	None	Cure

*PR* Present report, *N/A* Not available, *G* Gravidity, *P* Parity, *CS* Cesarean section, *VD* Vaginal delivery, *IVIg* Intravenous immunoglobulin, *SSI* Surgical site infection, *MSSA* Methicillin-susceptible *Staphylococcus aureu*s, *MRSA* Methicillin-resistant *Staphylococcus aureus*, *S. aureus Staphylococcus aureus*, *IV* Intravenous, *TSST-1* Toxic shock syndrome toxin-1, *SLE* Systemic lupus erythematosus, *N/A* Not available

**Fig. 2:**
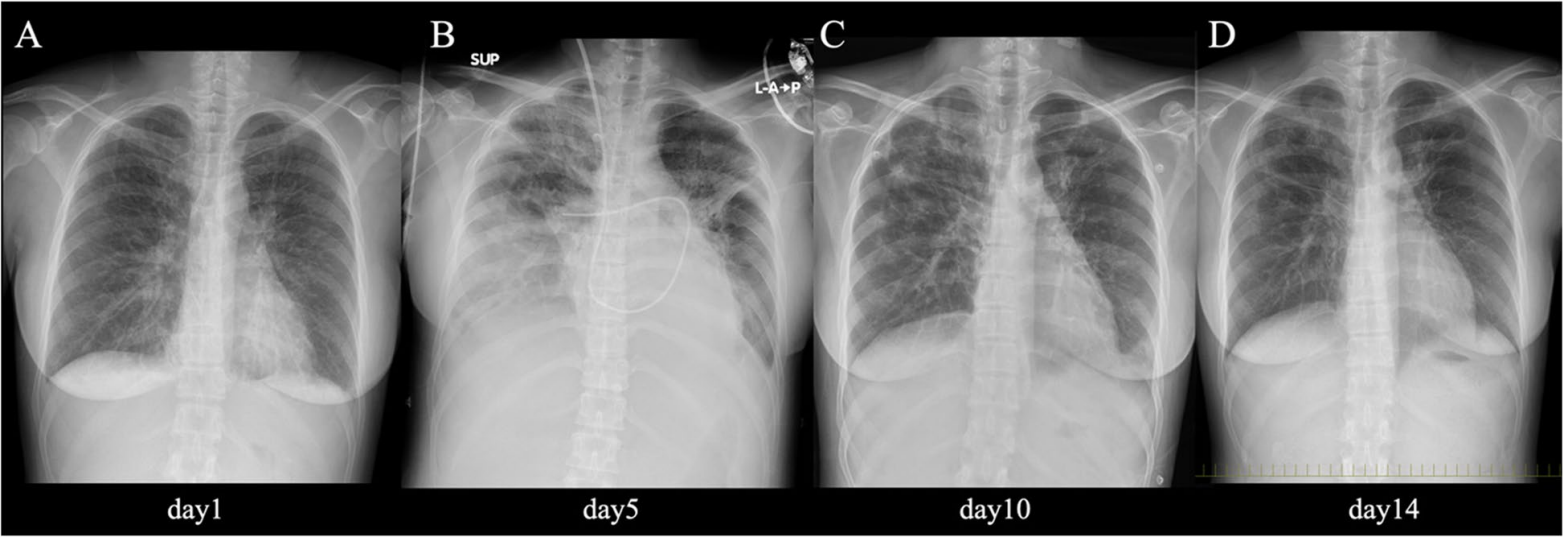
Changes in chest radiographs during hospitalization. **A** Day 1 chest X-ray (Upright position) revealed pulmonary vascular congestion, peribronchial cuffing, fluffy alveolar opacities, and Kerley B lines in both lungs. **B** Day 5 chest X-ray (Supine position) showed consolidation in both lung fields was markedly widened, and pleural effusion tended to increase, indicating exacerbation of pulmonary edema. The patient's respiratory failure worsened, and a Swan-Ganz catheter was inserted. **C** Day 10 chest X-ray (Upright position) showed the degree of cardiac enlargement was reduced, the enhancement of pulmonary vascular shadows was lessened, and pleural effusions decreased. **D** Day 14 chest X-ray (Upright position) showed that consolidation in both lung fields further improved, and the bilateral pleural effusions disappeared

**Fig. 3:**
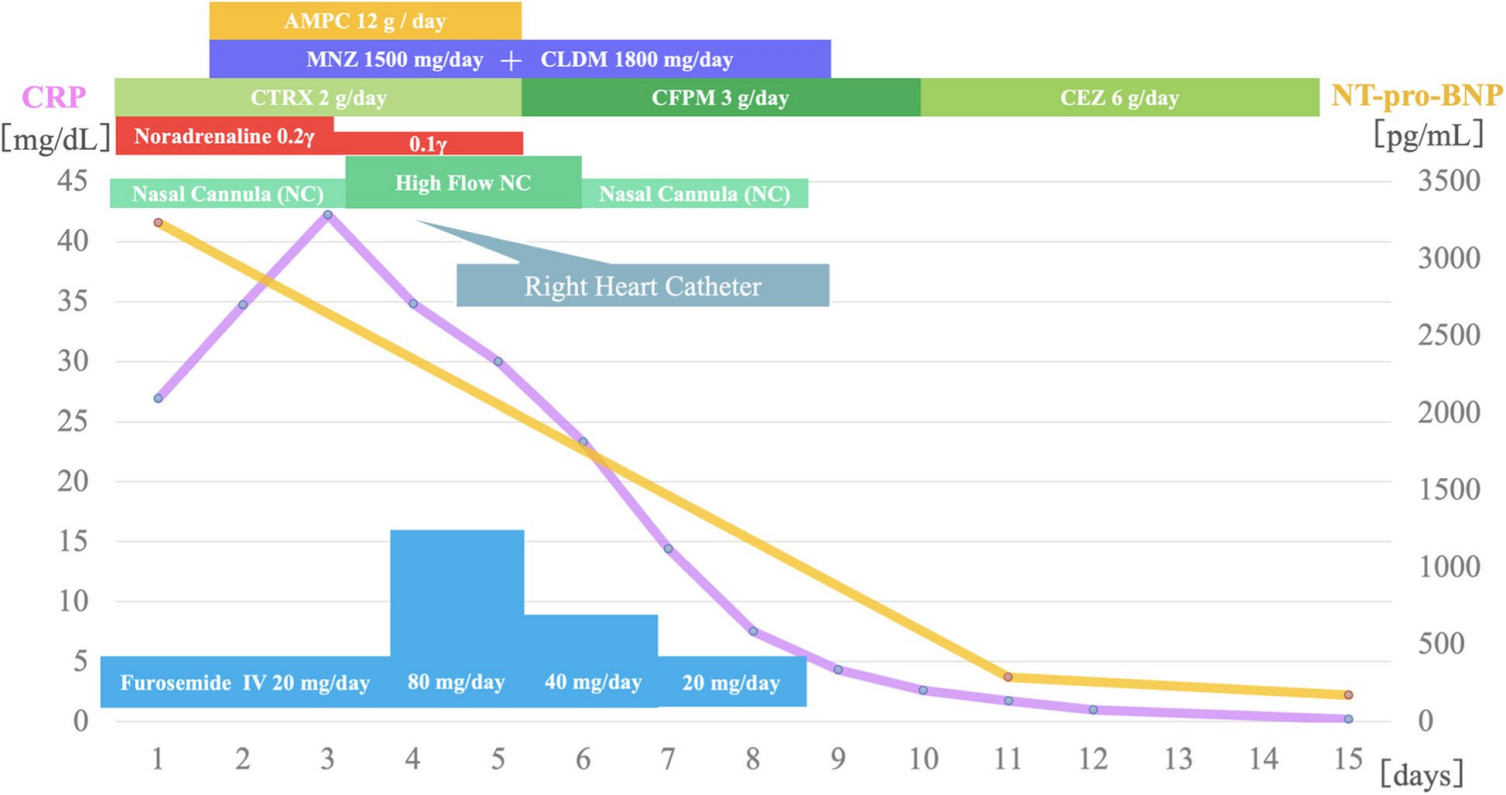
Clinical course timeline. CRP, C-reactive protein; NT-pro-BNP, N-terminal pro-brain natriuretic peptide; AMPC, amoxicillin; MNZ, metronidazole; CLDM, clindamycin; CTRX, Ceftriaxone; CFPM, Cefepime; CEZ, cefazolin; NC, Nasal Cannula; IV, intravenous

We empirically administered 2 g of intravenous (IV) ceftriaxone every 24 h and metronidazole 500 mg IV every 8 h. We added 2 g of IV ampicillin every 4 h for the possibility of streptococcal toxic shock syndrome (STSS) caused by β-hemolytic streptococci and 600 mg of IV clindamycin every 8 h for its inhibitory effect on toxin synthesis. Percutaneous drainage and washing of the perineal wound were performed daily after admission. In addition, furosemide 20 mg/day was started intravenously, and two units of packed red blood cells were administered. Cultures obtained from the perineum grew only MSSA. Pharyngeal cultures, blood cultures, and urine cultures were negative. Changes in chest X-ray over time during hospitalization are shown in Fig. 2. On the fourth day of admission, chest radiography showed enhanced pulmonary vascular shadows, and respiratory status deteriorated. We performed right heart catheterization to differentiate between worsening heart failure and acute respiratory distress syndrome (ARDS). It revealed a cardiac index of 4.2 L/min/m^2^, pulmonary artery wedge pressure of a28/v24/24 mmHg, mean pulmonary artery pressure of 36 mmHg, and body vascular resistance of 422.2 dyne-sec/cm^−5^, findings consistent with high flow heart failure and low peripheral vascular resistance associated with infection, so we introduced a high-flow nasal cannula. The furosemide dose was increased to 80 mg/day. On the eighth day after admission, the patient developed diarrhea with negative *Clostridioides difficile* culture, toxin, and antigen tests. On the 10th day of admission, TSST-1 was positive and this patient had a fever, rash, desquamation, and hypotension, as well as diarrhea, vaginal hyperemia, and liver dysfunction, so we diagnosed TSS caused by MSSA according to the CDC 2011 Case Definition of Toxic Shock Syndrome (other than Streptococcal) [4]. On the same day, antimicrobial therapy was changed to 2 g of IV cefazolin every 8 h and completed for a total of two weeks of treatment. Laboratory tests showed that CRP peaked at 42 mg/dL on the third day of admission and then gradually decreased, and NT-pro-BNP also declined to 171.1 pg/mL at the time of discharge (Fig. 3). As shown in Table 1, subsequent echocardiographic findings also showed a trend toward improvement in left ventricular diastolic dysfunction, and TRPG decreased to the normal range. Her respiratory condition improved quickly, and the patient’s fingers and face desquamated on the eleventh day. The patient was clinically stable and discharged on hospital day 15, and no recurrence of heart failure or infections was observed during 24 months of follow-up.

## Discussion and conclusions

TSS is a life-threatening disease; therefore, early diagnosis and appropriate treatment are required. Half of the reported staphylococcal TSS cases are non-menstrual [7, 8]. The causes of non-menstrual related TSS include vaginal and cesarean deliveries, surgical and wound infections [9, 10], post-miscarriage [11], mastitis [12], bacterial laryngotracheitis [13], sinusitis [14], arthritis [15], enteritis [16], and skin lesions [17]. Among them, TSS associated with surgical sites or wounds is more difficult to diagnose, because it may occur in the absence of obvious signs of infection in the wound [18]. In a previous report, identifying the primary source was difficult in approximately 40% of cases, indicating that it is not easy to identify the local findings that cause infection [19]. Current diagnostic criteria for TSS, as published by the CDC, include fever, rash, desquamation, hypotension, and multisystem involvement, including gastrointestinal, muscular, renal, and hepatic involvement [4]. In the current case, fever, rash, desquamation, and hypotension were all present, and, in terms of the multisystem involvement, diarrhea, vaginal hyperemia, and elevated liver enzyme levels were observed. In addition, the identification of *S. aureus* from the lochia, incisions, and the exclusion of other diseases led to the diagnosis of TSS. However, only the minor redness of the trunk and mild vaginal inflammation were seen at the time of admission, and our patient mainly showed signs of heart failure, indicating the difficulty of recognizing the primary source and the importance of identifying it.

**Fig. 4:**
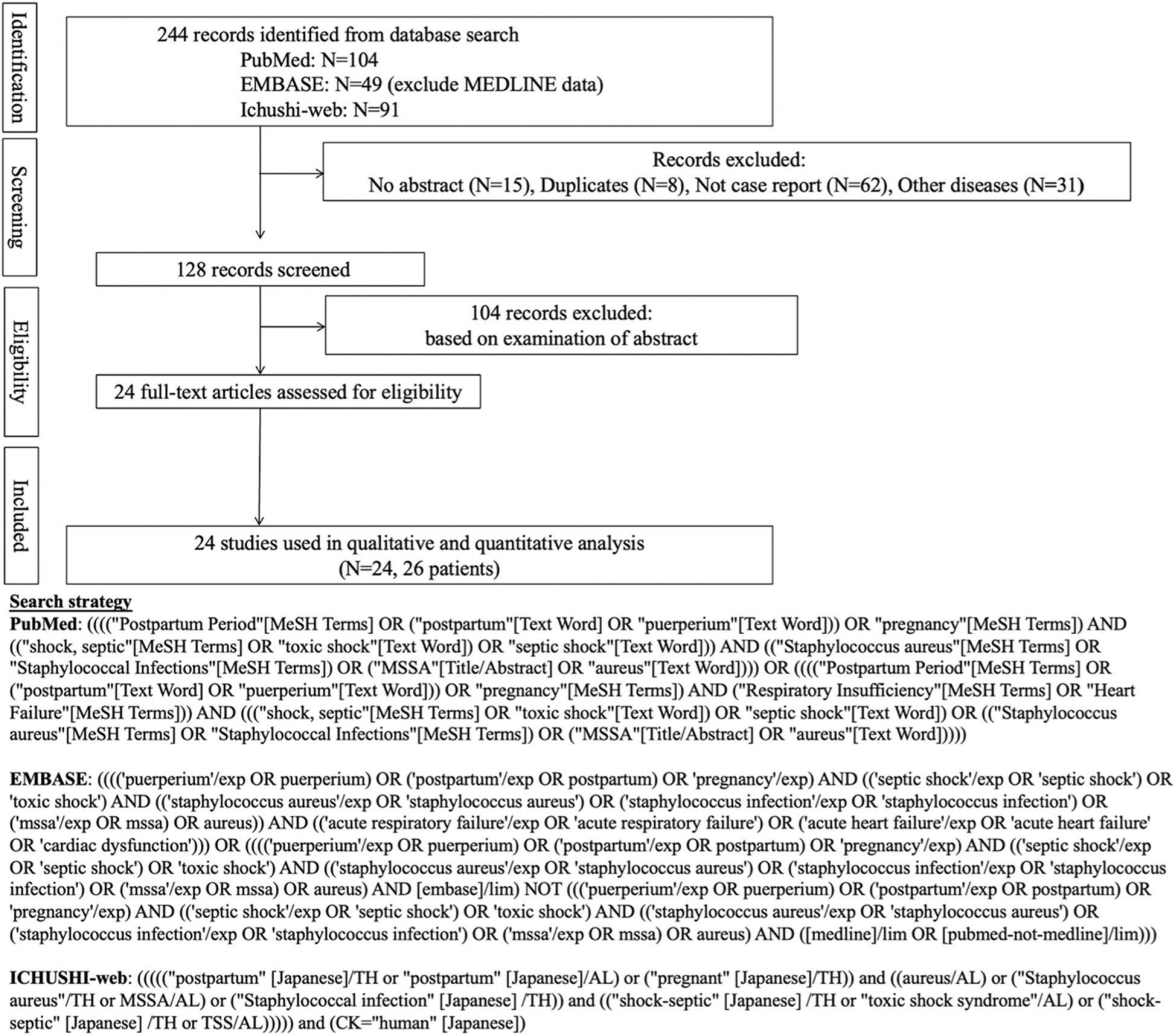
Flow diagram and search strategy for a systematic literature review

We conducted a systematic review using MeSH terms and made database including authors information, titles, abstracts, and languages by using them. Three researchers independently searched PubMed, EMBASE, and Ichushi-Web from their inception to August 1, 2022, to review and extract relevant cases. The respective search strategies for PubMed, EMBASE, and Ichushi-web are described in Fig. 4; 24 articles were retrieved, two of which describe two cases of postpartum TSS [3, 12, 20–41]. As a result, we found 27 cases of TSS due to *S. aureus* during the postpartum period. The summary and detailed information on the clinical characteristics of the 27 cases, including our case, are shown in Table 1. The median age of the patients was 28 years. Common symptoms were fever in 100% of patients (27/27 cases), followed by gastrointestinal symptoms (nausea, vomiting, diarrhea, and abdominal pain) in 74% (20/27 cases), and rash (erythema) in 52% (14/27 cases). The onset of TSS often occurs approximately 8 days after delivery, with a minimum of the same day and a maximum of 28 days. Vaginal delivery accounted for 19 cases, cesarean section accounted for 7 cases, and one case was unknown. The causative organisms were methicillin-resistant *Staphylococcus aureus* (MRSA) in 44% (12/27 cases) and MSSA in 48% (13/27 cases) of cases. No data were available for MSSA or MRSA in 2/27 patients (7%). The portal of entry was diverse, with 15/23 cases (65%) of endometritis and perineum, followed by 5/23 cases (22%) of mastitis and 3/23 cases (13%) of surgical site infection (SSI) after cesarean section. As for superantigens, TSST-1 was positive in 11/27 cases (41%), and enterotoxins B and C were positive in 1 case (4%) and 4 cases (15%), respectively. Complications of heart failure were observed in 2 cases, including ours. In our patient, heart failure preceded the onset of TSS, but in the other case, cardiomyopathy or heart failure was observed 9 days after the onset of TSS. The median duration of antimicrobial therapy was 12 days, and no fatalities were reported in any of the cases.

Heart failure (HF) is a clinical syndrome caused by structural or functional cardiac abnormalities with symptoms or signs supported by objective evidence of elevated natriuretic peptide levels and pulmonary or systemic congestion [42]. In our literature review, we found several cases of respiratory insufficiency due to ARDS [24, 33], but there has been only one case of heart failure [20]. In this case, heart failure appeared approximately ten days after the onset of TSS. The development of congestive heart failure secondary to cardiomyopathy was suspected, which is different from our case, where our patient's initial presentation included acute heart failure and our case differs from the previously reported case in that the left ventricular systolic function was preserved. In any case, clinicians must keep in mind that patients with TSS can present solely with heart failure, which can be improved with appropriate therapeutic intervention.

Regarding TSS with cardiac complications, one report suggests that TSS is associated with reversible cardiomyopathy in rats, suggesting that TSST-1 may directly interfere with cardiac function [43]. In addition, heart failure as a cardiac complication of TSS has been reported in cases of tampon use; myocardial damage, ischemic changes, and multiple subendocardial microinfarcts associated with shock have been considered as possible mechanisms [44]. On the other hand, septic myocardial damage is called septic cardiomyopathy (SICM), reported as reversible myocardial damage by Parker et al. in 1984, but there is no current clear definition [45]. Sepsis is defined as a serious condition where infection causes severe organ damage [46], and SICM, as a sepsis-related manifestation, contributes to circulatory collapse, which, if not adequately treated, can lead to multiple organ dysfunction and increase the mortality rate by two to three-fold [47, 48]. Elevated blood troponin levels have been reported to be a poor prognostic factor in the setting of heart failure, as it is a marker of myocardial damage [49]. SCIM caused by TSS may be due to functional or structural changes in cardiomyocytes and myocardial microcirculatory disturbances caused by excessive cytokines [47, 48]. In our case, the NT-pro-BNP level was markedly elevated, accompanied by high troponin levels and pleural effusion, and physical examination showed edema of the legs and distended jugular veins, suggesting that such myocardial damage was caused by the toxin. In addition, a cardiac index of this case was 4.2 L/min/m^2^ and it was consistent with high output heart failure. Its causes include anemia, thyrotoxicosis, physiological changes (pregnancy, fever, infection, etc.), and congenital diseases [50], and, in our case, anemia, infection, and post-pregnancy status may have led to high output heart failure. Although the echocardiogram did not show any impairment of cardiac contractility, clinicians should consider the possibility that SCIM may be a possibility nonetheless. As for the other feasible diagnoses that would fit the clinical presentation, viral myocarditis was ruled out via blood tests, and perinatal cardiomyopathy was assumed to be a diagnosis of exclusion in the absence of any other cause of heart failure. In the present case, the left ventricular ejection fraction was preserved at approximately 60%, which is not consistent with peripartum cardiomyopathy, given that left ventricular systolic dysfunction was unremarkable [51–53].

It should be noted that hemodynamics during pregnancy and puerperium may be different from those of a normal state. During pregnancy, several physiological adaptations usually take place to satisfy the increased metabolic requirements of the mother and fetus and maintain placental perfusion; these changes revert back to a normal state about 2 weeks after delivery [54]. That is, after delivery, the increased cardiac output of pregnancy drops to 10%, and systemic vascular resistance also increases rapidly and returns to pre-pregnancy levels within 2 weeks [55, 56]. Therefore, in this case, the decrease in peripheral vascular resistance and increase in cardiac output detected by the Swan-Ganz catheter at 2 weeks post-pregnancy cannot be explained by the pregnancy itself.

Regarding the toxins in TSS, detecting superantigens such as TSST-1 has been beneficial [57] and was identified in approximately 40% of cases in this review. Non-menstrual staphylococcal TSS is not associated with all superantigens and is recognized in approximately 50% of cases [58, 59], similar to the one in this report. TSST-1 is known to be a superantigen essential for the pathogenesis of TSS. Superantigens may influence the pathogenesis of cardiovascular and vascular diseases associated with *S. aureus* infections [57]. Although other enterotoxins A, B, C, D, E, and H have been reported [60, 61], TSST-1 has a higher mucosal penetrating ability than enterotoxins B and C, and the amount of toxins produced by TSST-1 alone is thought to be much higher than that required to cause disease and may cause serious complications [57]. In our review, TSST-1 positivity was 20% (2/10 cases) for MSSA strains and 53% (8/15 cases) for MRSA strains; therefore, TSST-1 positivity tended to be higher in MRSA strains, which is consistent with previous reports [62–64]. Thus, toxin positivity may be higher in MRSA strains, but the direct relationship with prognosis is not clear, as all cases in our review successfully recovered. However, it is necessary to consider the limitation that not all case reports have reported toxin results.

In terms of treatment, it is essential to intervene as soon as possible and thoroughly search for the focus of the infection. In this case, although the superficial infection was minimal, we detected purulence on the inside of the perineal incision, and drainage was immediately performed. The tissue infection may be much more widespread than originally thought, and so, even if the local inflammatory findings are mild, it should be considered a potential source of infection [65]. Inappropriate initial antimicrobial therapy may even increase mortality in intensive care patients with severe sepsis and septic shock [66, 67]. There are no randomized controlled studies suggesting antimicrobial selection for toxic shock syndrome, and evidence based on case series recommends initiating anti-staphylococcal treatment as empiric therapy [1, 68]. One guideline recommends that the initial antibiotic should include vancomycin, linezolid, daptomycin, telavancin, or ceftaroline for MRSA coverage if the institution has high proportion of MRSA infection or the patient is at high risk for bacterial resistance [69]. Additionally, they recommend the use of one of the following antibiotics if we consider Gram-negative and anaerobic bacteria; (1) piperacillin tazobactam, (2) carbapenem, or (3) ceftriaxone and metronidazole. Clindamycin is also recommended in toxic shock syndrome because limited in vitro data have shown that clindamycin in combination with standard therapy in TSS can be expected to inhibit the production of multiple exotoxins [70, 71]. Various studies have shown that clindamycin is a major protein synthesis inhibitor antibiotic active against *S. aureus* and reduces the production of bacterial toxins, and combination therapy, including standard treatment, is considered [72]. De-escalation to cloxacillin or nafcillin or cefazolin, and clindamycin is recommended after the causative organism is identified as MSSA [65]. At the present time, there is a lack of clinical studies on the appropriate duration of antimicrobial therapy for TSS due to *S. aureus* infection. This review shows that the median duration of therapy in all cases was approximately 12 days. This review also indicates that 5 of the 15 cases (33%) with MRSA strains were started on anti-MRSA therapy from the beginning, 9 cases (60%) needed to be changed to anti-MRSA therapy during treatment, and one case (7%) was infected with MRSA but completed treatment with non-anti-MRSA therapy. Although there have not been many reports of TSS due to postpartum MRSA, routine anti-MRSA therapy is not recommended [26]. The possibility of infections in pregnant women during hospitalization after delivery has been reported to be 0.11% [73], and it is essential to consider the risk factors for MRSA infection in each healthcare setting. Therefore, anti-MRSA therapy should be evaluated based on each patient’s risk factors or in cases where MRSA colonization has already been observed in the past [74]. Regarding prognosis, all cases included in our review had a surprisingly good prognosis, suggesting a trend toward a better prognosis compared with the mortality rate in non-menstrual related TSS, which is approximately 20% [75]. However, the number of cases is insufficient for general conclusions, and more cases are expected in the future.

TSS due to MSSA during the postpartum period is not common, but the diversity of symptoms requires appropriate diagnosis and early intervention because of the potential for increased mortality if the diagnosis is delayed. To the best of our knowledge, this is the first systematic review of non-menstrual related TSS due to *S. aureus* during the postpartum and the case report in which the initial presentation was pure heart failure. The strength of this review is that in addition to focusing on heart failure, a complication of TSS, it provides a clinical course and prognosis. This case report also offers new insights into the development of TSS under the guise of heart failure, which is essential for health care professionals who may generally be involved in TSS treatment. The multifaceted perspective provided by obstetrics and gynecology specialists, infectious diseases specialists, as well as cardiologists, from the beginning of hospitalization enabled early therapeutic intervention (appropriate antimicrobial agents, wound drainage, and control of heart failure). This case report teaches us that the clinical course of TSS can include a presentation of sole heart failure and suggests that the possibility of TSS should be kept in mind in cases presenting with heart failure during the postpartum phase.

## Supplementary Information

**Additional file 1.** The echocardiographic four-chamber image on admission. The echocardiographic four-chamber image is shown in Video 1. Transthoracic echocardiography revealed unremarkable left ventricular size and wall thickness with a normal ejection fraction. It corresponds to having intermediate left ventricular diastolic dysfunction.

**Additional file 2.** The echocardiographic image of the inferior vena cava on admission. The dilated inferior vena cava (IVC) is shown in Video 2. The IVC was dilated to 21.7/18.4 mm (dilation on inspiration and expiration), indicating decreased respiratory variability.

## Data Availability

Detailed information about the case in this report is available from the hospital information system upon request. A search formula for systematic reviews was clearly indicated, and the literature search is available using this search formula. Our Excel file dataset extracted from this search is available from the Corresponding Author upon request.

## Abbreviations

TSS: Toxic shock syndrome
TSST-1: Toxic shock syndrome toxin-1
MSSA: Methicillin-susceptible *Staphylococcus aureus*
TTE: Transthoracic echocardiography
CT: Computed tomography
STSS: Streptococcal toxic shock syndrome
ARDS: Acute respiratory distress syndrome
MRSA: Methicillin-resistant *Staphylococcus aureus*
HF: Heart failure
SICM: Septic cardiomyopathy
